# MacIntyre, Weber and institutional logic*s*

**DOI:** 10.3389/fsoc.2022.983190

**Published:** 2022-10-28

**Authors:** Irene Chu

**Affiliations:** Newcastle University, Newcastle upon Tyne, United Kingdom

**Keywords:** MacIntyre, Max Weber, institutional logics perspectives, morality & values, practice

## Abstract

This paper explores “what more and what else” MacIntyre's concepts can contribute, specifically as applied to neoinstitutional theory and especially institutional logics. Drawing on the common influence of Max Weber's work as further developed by Friedland, MacIntyre's concept of *eudaimonia* being furthered by the pursuit of internal goods supported by external goods is used to develop a typology of goods. This typology is then deployed to show how the differing institutional logics of, for example, the market and the family have differing rationalities with differing emphases on internal and external goods, and consequently differing moral content. A simple picture of the market economy is then developed to show how such MacIntyrean concepts can be used to address the critique of a lack of morality in neoinstitutional theory. Conversely, the analytical framework provided by the institutional logics perspective is used to show how MacIntyrean concepts can be applied practically in a way that provides an interesting perspective on the current world.

## Introduction

Neo-institutional theory and its later derivatives such as the institutional logics perspective have become dominant within the field of organization studies (Alvesson and Spicer, 2019). Indeed this success has become an area of concern for some who criticize its “hydra-like” (Willmott, 2018) and “juggernaut” nature (Lok, 2019). This field of study may seem to be distant from MacIntyre's moral philosophy, but there are perhaps surprising similarities between the two. This paper will explore these similarities and then, hopefully fruitfully, investigate what each field can learn from the other.

The influence of Max Weber on both MacIntyre and institutional logics is evident. MacIntyre acknowledges this at the end of his critical argument in *After Virtue*—“The contemporary vision of the world, so I have suggested, is predominantly, although not perhaps always in detail, Weberian” (MacIntyre, 2007, p. 109). Likewise Friedland, who can be considered to be one of the founders of institutional logics, describes how Weber “a century before, laid out a way to conceptualize institutional logics” (Friedland, 2013b, p. 4).

Whilst not denying that there may be ontological and epistemological concerns around bringing MacIntyrean notions together with those from institutional logics, I will argue that such concerns can be allayed and that each area can contribute to the other. For example, a major criticism of neo-institutional theory is its lack of an adequate appreciation of value and morality^1^,^1^. I contend that this can be addressed by using MacIntyre's notion of internal, common and external goods to develop a typology of goods, each with different moral content. The practices found in each institutional logic generate both internal and external goods, but it is the internal goods which are important for Weber's value rationality, driven by Friedland's institutional substances, and which contribute to MacIntyre's *eudaimonia*. It is the virtues which enable the distinguishing between internal and external goods and thus acknowledgment of the balance needed between the two. In this way, MacIntyre's virtue ethics can introduce morality into institutional logics and address the criticism of their value neutrality referred to above.

In the opposite direction, concepts from institutional logics can also be fruitfully applied to MacIntyre's works. The concept of internal goods can be strengthened by Weber's value rationality and Friedland's institutional substances. Also, institutional logics' typology of the social order being composed of individual logics, together with its analytical separation of the micro-, meso- and macro-levels, can be combined with MacIntyrean concepts to produce a simplified but illuminating view of the market economy. In this way, the author hopes to demonstrate not only how the two areas can benefit each other but also how the application of MacIntyrean concepts to the analytical framework provided by the institutional logics perspective may illustrate “what more and what else” can result from MacIntyre's work.

## Reviewing the roots

### MacIntyre

The importance of MacIntyre as a philosopher of virtue is well-established (Moore, 2015, p. 101), as evidenced by the fact that, after Aristotle, he is the most widely cited writer in the area of virtue ethics in business (Ferrero and Sison, 2014). He is extensively read outside philosophy (Beadle, 2017; Beadle and Moore, 2020), for example in business ethics (e.g., Beadle, 2002; Beadle and Moore, 2006; Bernacchio, 2018; Sinnicks, 2019, 2020; Chu and Moore, 2020) and organizational thought (e.g., Anthony, 1986; Alvesson and Willmott, 1992; Mangham, 1995; Du Gay, 1998, 2000; Tsoukas, 2018) including strategy-as-practice (Tsoukas, 2017) and leadership theory (Crossan et al., 2017). For present purposes, our focus is on the concepts presented in *After Virtue* (MacIntyre, 2007), mainly his three level framework of the virtues but especially internal and external goods generated by practices housed within institutions.

Internal goods involve both the excellence of products of the practice and, more importantly, the “perfection” of the practitioner (MacIntyre, 1994, p. 284; 2007, p. 189–190), where ‘perfection' contributes to *eudaimonia*, the notion of human flourishing. Thus, internal goods are “bound up with an authentically experienced emotional engagement with work” (Fisher and Byrne, 2012, p. 80), the satisfaction and rewards of a job well-done. Examples include health care (Fisher and Byrne, 2012), heritage curatorship (Townley, 2002), architecture (Moore, 2017), and finance (e.g., Robson, 2015; Rocchi et al., 2020). In contrast, external goods such as money, power, status and success are instrumental rather than intrinsic goods. They are means to further ends and so should be primarily valued and pursued for the sake of the internal and common goods which they enable. MacIntyre stresses that it is the internal goods which are fundamental for human flourishing but they have typically become neglected in the pursuit of external goods. Internal and external goods have been linked with Weberian value and instrumental rationality, respectively (Townley, 2002; Fisher and Byrne, 2012), so internal goods can be considered to function partly as the reward of the individual as a result of their value rationality driven action, the positive feelings people experience when in harmony with institutions “by enjoying a fit with societal values” (Weik, 2019, p. 329). This has been similarly described by Higgins (2010) in terms of a “moral phenomenology,” an ephemeral experience of being in contact with the distinctive mode of being which the practice affords. However, these differences between internal and external goods may not be readily apparent and, indeed, are obscured by the tendency to focus on external goods. For MacIntyre, it is the virtues which enable the recognition and achievement of internal goods as well as their prioritization over external goods, allowing individuals to distinguish between “what any particular individual at any particular time takes to be a good” and “what is really good” (MacIntyre, 2007, p. 150).

### Neo-institutional theory

For readers less familiar with the institutional logics perspective, it may be instructive to quickly review its conceptual origins. New institutionalism, or neoinstitutional theory as it generally became known, is now acknowledged to have started with a series of seminal works from the 1970s onwards. The most important of these include the works of Meyer and Rowan (1977), DiMaggio and Powell (1983) and Friedland and Alford (1991), each of which demonstrate the influence of Weber. Indeed, the importance of Weber for later institutional theory can be seen by the fact that more contemporary institutional theorists cite him as a significant influence than any other early theorist (Scott, 2014). For example, following on from Weber's notion that organizational structure is influenced by concerns of how organizations gains legitimacy, Meyer and Rowan (1977) investigated how the formal structures of schools and associated artifacts, such as organization charts, goals and policies, were decoupled from their core educational activities. Instead, they were more to do with conformity to rationalized institutional rules, described as myths and ceremony, and conformity to these rules was felt to be needed to gain legitimacy, resources and stability and so enhance survival prospects. Building similarly on Weber, this time his concept of the iron cage resulting from bureaucracy, DiMaggio and Powell (1983) researched why organizations took homogeneous forms where more diversity might be expected. They proposed that the observed structural isomorphism was not the result of bureaucratisation but structuration caused by three mechanisms, which they labeled coercive, mimetic, and normative. This recognition of differing sources of pressures for isomorphism originating in different areas of the institutional environment, together with the concept of organizational fields, resulted in their 1983 paper “The Iron Cage Revisited: Institutional Isomorphism and Collective Rationality in Organizational Fields” being well-cited and highly influential (Thornton et al., 2012, p. 25).

Together with a paper by Scott and Meyer (1983), who proposed that all organizations are shaped by both technical and institutional influences but that these two have unequal effects on different organizations, these early works were reprinted by Powell and DiMaggio (1991) as foundation works in their *New Institutionalism in Organizational Analysis*. This ‘orange volume' is considered to be a seminal work in neoinstitutional theory (Scott, 2014) and marks a defining point between old and new institutionalism. In it, Powell and DiMaggio (1991) summarized the differences between these two and contended that the root of these lies in different notions of the cultural or cognitive foundations of institutionalized behavior. Thus for old institutionalists, typified by Selznick (1957), salient cognitive forms were values, norms and attitudes whereas taken for granted scripts, rules and classifications are more important for new institutionalists. Instead of institutionalization occurring at the organizational level, with organizations becoming “infused with value” (Selznick, 1949), new institutionalism sees institutionalization occurring in the environment of organizations, often at field level with institutions being macro-level abstractions, “rationalized and impersonal prescriptions” (Meyer and Rowan, 1977, p. 343) or shared typifications (Berger and Luckmann, 1966/1991). However, together with this greater level of abstraction, it is often felt that neoinstitutionalism has downplayed the role played by values to such an extent that there have been calls for more emphasis to be placed in this area (Friedland, 2012; Moore and Grandy, 2017).

However, for present purposes, the most relevant work contained in the orange volume is that by Friedland and Alford (1991)—“Bringing society back in: Symbols, practices and institutional contradictions.” In this work, they argued that the concept of society was being neglected in social sciences with too much emphasis being given to the utilitarian individual and to power-oriented organizations, with the former being prioritized by agency theory, rational-actor models and public-choice theory. However, such “notions of individual choice and agency are contingent modern products. …People in many of these [non-Western] societies are less likely to conceptualize themselves [as] individuals independently of the roles they occupy and the contexts in which they are situated” (Friedland and Alford, 1991, p. 239). Western individualism has been shaped by the emergence of capitalism, the state, democracy, the nuclear family and Christianity, and theories of society need to take these influences into account. Furthermore, any such theories also need to work at three levels of analysis, at those of the individual, the organization and the institutional, both symbolic and material, with each level containing aspects of competition and interdependency. In order to bring these three levels together, they posited that the institutional level is crucial in linking symbols and practices but that this needs a new definition of institutions:

“Institutions are supraorganizational patterns of human activity by which individuals and organizations produce and reproduce their material subsistence and organize time and space. They are also symbolic systems, ways of ordering reality, and thereby rendering experience of time and space meaningful.” (Friedland and Alford, 1991, p. 243)

Building on this definition, Friedland and Alford (1991) were able to propose a more complete model which was able to address deficiencies of contemporary neoinstitutional theories in areas such as interests, power and agency. They argued that theories of the classical scholars Marx, Durkheim and Weber were all based on assumptions that the world was built “from the ground up,” that is on the real material structure of society, giving rise to materialist-idealist or base-superstructure dichotomies. However, cultural symbols can be both sources and carriers of individual behavior and so these classical models require reconstruction to reflect the centrality of the symbolic in social life. To this end, they proposed:

“each of the most important institutional orders of contemporary Western societies has a central logic—a set of material practices and symbolic constructions—which constitutes its organizing principles and which is available to organizations and individuals to elaborate” (Friedland and Alford, 1991, p. 248)^2^.

^2^

### Institutional logics

However, this concept of multiple institutional logics lay dormant for almost a decade until it was picked up by Thornton and Ocasio (1999) who, together with Lounsbury, developed it further into a metatheoretical framework now known as the institutional logics perspective (Thornton et al., 2012). They describe that their aim in doing this was to transform neoinstitutional theory, both building on its strengths in the areas of how the macro-environment shapes organizations and addressing its weaknesses around agency, the micro-foundations of institutions, institutional heterogeneity, and change. The result was “a new approach that incorporates macro-structure, culture, and agency, through cross-level processes (society, institutional field, organization, interactions and individuals) that explain how institutions both enable and constrain action” (Thornton et al., 2012, p. vi). They propose seven institutional logics—the family, community, religion, state, market, profession and corporation—and their definition of an institutional logic is as follows:

“the socially constructed, historical patterns of cultural symbols and material practices, including assumptions, values and beliefs, by which individuals and organizations provide meaning to their daily activity, organize time and space and reproduce their lives and experiences” (Thornton and Ocasio, 1999, p. 84).

**Figure 1:**
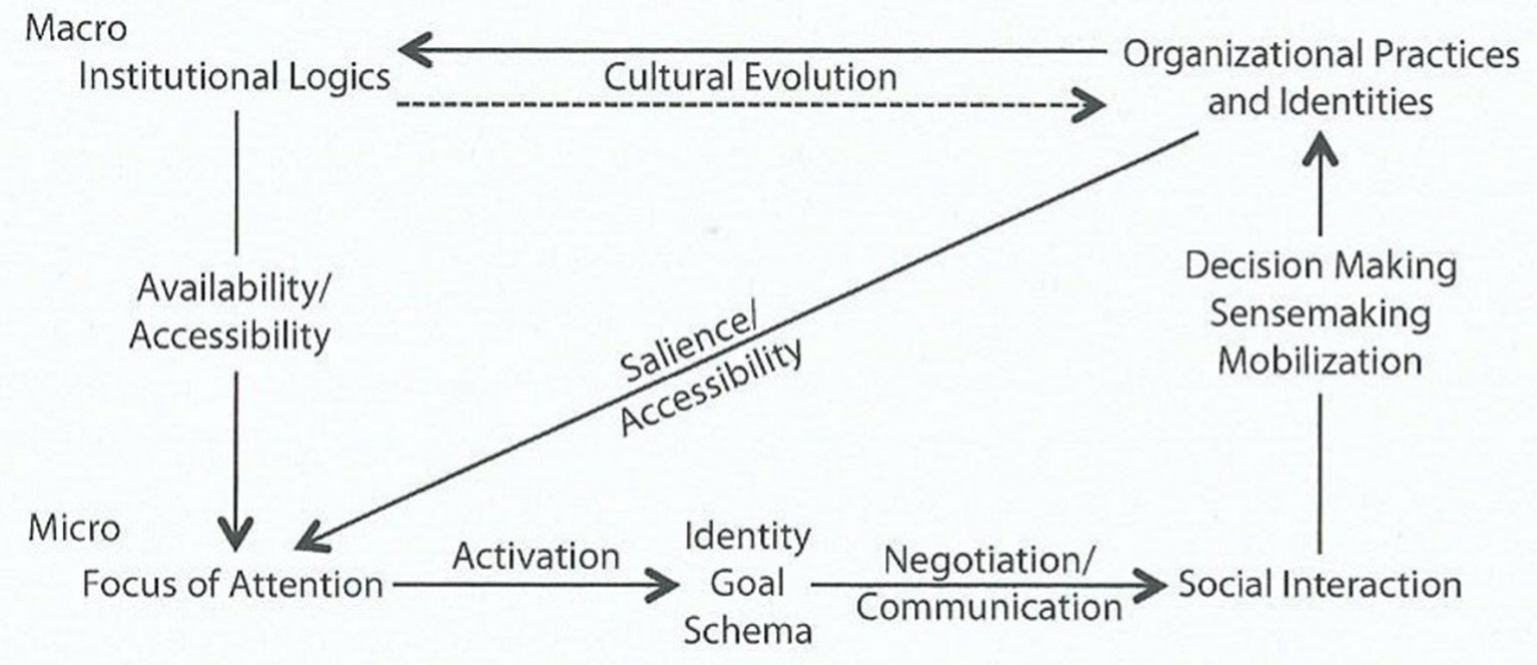
A cross-level model of institutional logics. Source: Reproduced from Thornton et al. (2012).

Perhaps the most illuminating model from the institutional logics perspective is the cross-level model shown in Figure 1 below. This describes how institutional logics at the macro-level influence individuals at the micro-level, depending on the former's availability, salience, and accessibility. At the micro-level, this influence triggers individuals' social identities, goals and schemas and then, through interacting with each other, the individuals collectively produce social practices and structures, including organizations. These practices then undergo a process of cultural evolution resulting in selective retention, and they influence the development and stability of institutional logics, again at the macro-level.

This model provides a powerful framework for interpreting observations of both the material and symbolic made at different levels of society—at the individual, organizational and institutional logics levels. Furthermore, it is also a “model of social actors whose identities and goals are not only embedded in institutional logics, but also situated in organizational practices” (Thornton et al., 2012, p. 102). This leads us to perhaps the most obvious concept linking MacIntyre and the institutional logics perspective—the centrality of practices. MacIntyre's seminal description of practices is well-known but given again here to aid the reader:

“Any coherent and complex form of socially established cooperative human activity through which goods internal to that form of activity are realized in the course of trying to achieve those standards of excellence which are appropriate to, and partially definitive of, that form of activity, with the result that human powers to achieve excellence, and human conceptions of the ends and goods involved, are systematically extended” (MacIntyre, 2007, p. 187).

This can be contrasted with that from the institutional logics perspective—Thornton et al. (2012) state that “practice refers to forms or constellations of socially meaningful activity that are relatively coherent and established (see, e.g., MacIntyre, 1981)” (Thornton et al., 2012, p. 128)' and the citation of MacIntyre is obviously noteworthy. An explicit distinction between practices and activities is also made, with activities referring to mundane behaviors which are generally devoid of social meaning and are not informed by wider cultural beliefs. “Pounding a nail” is an activity whereas carpentry is a practice providing order and meaning to a set of otherwise banal activities^3^.

However, it is evident that the institutional logics perspective definition lacks the nuances of value included in MacIntyre's definition and the critique from Friedland (2012) in his review of Thornton et al. (2012) is important. Whilst noting that the omission of value might be a desire to avoid the “normative legacy” of old institutionalism, he argues that “value is central to an institutional logic: a presumed product of its prescribed practices, the foundation stone of its ontology, the source of legitimacy of its rules, a basis of individual identification, a ground for agency, and the foundation upon which its powers are constituted” (Friedland, 2012, p. 585). Whilst acknowledging that there is a link to the concept of legitimacy presented in the institutional logics perspective, it is clear that the importance of this lack of value should be emphasized more strongly. In this way, Friedland contends that “an institutional value founds the ontology of the central object or state of being, around which normatively enforced practices are organized through constellations of roles and hence constitute the resources through which powers are afforded” (Friedland, 2012, p. 586).

Furthermore, the micro-foundations of institutional logics lack feeling and passion, a factor which “Weber once attended in his treatment of the “value spheres,” and we appear to have forgotten (Weber, 1920/1958). Aristotle recognized this long ago when he pointed to virtue as a direction of desire, “desiring reason,” without which practical rationality vis-a-vis justice could not function (MacIntyre, 1988, p. 136–137; Friedland, 2012, p. 594) and also “To put it baldly, institutional logics depend upon love (Friedland, 2013a, 2014)” (Friedland, 2012, p. 594). For Friedland, each of the institutional logics is anchored by its own institutional substance akin to Aristotelian substantial form and each has its own value rationality driven by a “logical or teleological ‘consistency', a consistency that exercises a ‘power over man' (Weber, 1920/1958, p. 324)” (Friedland, 2013b, p. 16).

In his article “God, Love and Other Good Reasons for Practice: Thinking Through Institutional Logics,” Friedland (2013a) expanded this concept, which appeared in Weber's “Religious Rejections of the World and their Directions” (Weber, 1920/1958). This essay has been described as “a key text, perhaps the key text in Weber's entire corpus” (Bellah, 1999, p. 2) and Friedland also considered it to be “extraordinary” with Weber having “a century before, laid out a way to conceptualize institutional logics” (Friedland, 2013b, p. 4). Friedland claims that he “came late to Weber” (Friedland, 2013a, p. 4), but it is clear that Weber's description of the social world as a multiplicity of ‘value spheres' (religious, economic, political, aesthetic, erotic, and intellectual), sometimes also referred to as ‘life-orders', are remarkably similar to neo-institutional theory's institutional logics (Friedland and Alford, 1991; Thornton et al., 2012).

Weber differentiates between different types of social action—instrumentally rational, value-rational, affectual, and traditional—with instrumental rationality guiding actions where other people or objects are used as the means for the “actor's own rationally pursued and calculated ends” and value rationality being “determined by a conscious belief in the value for its own sake of some ethical, aesthetic, religious, or other form of behavior, independently of its prospects of success” (Weber, 1922/1978, p. 24–5). Friedland describes value-rationality as being located in each of the value-spheres, so that action within each is “oriented toward determinate, incommensurable, ultimate values: divine salvation in religion, aesthetics in art, power in politics, property in capitalist markets, erotic love, knowledge in science” (Friedland, 2013b, p. 5). Friedland also conceives the term ‘institutional substance' in this respect—“What Weber calls the ‘gods' of the value spheres I have termed institutional ‘substances,' the unobservable, but essential, ‘value' anchoring an institutional logic” and also “a substance is the metaphysical foundation of the institutional logic, which provides the *telos* of the subject, the basis of her identity” (Friedland, 2013b, p. 19 emphasis added).

The types of social action are also associated with Weber's types of rationality; Kalberg (1980) links value-rational action to substantive rationality and instrumentally rational action to practical and formal rationality, with different rationalization processes taking place in the different value-spheres (Kalberg, 1980, p. 1,150). Consequently, although value rationality is present in each of the spheres and so also in each institutional logic, instrumental rationality is more dominant in some of them, for example, in that of the market and the state (Friedland, 2013b, p. 21). Thus, it can be seen that Weber's value-spheres and institutional logics can be linked, with each logic being driven by types of social action associated with types of rationality. However, in the cause of clarity following the example of Friedland (2013b), the terms instrumental and value rationality will be used throughout the following discussion (in place of instrumentally rational behavior linked to practical or formal rationality and value-rational behavior linked to substantive rationality).

Brubaker (1984) considered rationality to be the uniting theme in Weber's works but contended that his ideas on rationality are not generally accessible because Weber did not systematize them and also since his voluminous work is generally approached in a piecemeal fashion. However, a central theme is the irreconcilable tension between formal and substantive rationality, which can be considered synonymous with instrumental and value rationality, respectively, as described above. He also contended that this tension exists because the institutional foundations of the modern economic order are morally and politically problematic, since they involve the “struggle of man against man” in the marketplace (Weber, 1922/1978, p. 93, 108) which is “an abomination to every system of fraternal ethics” (p. 636). Whilst generally unsympathetic to socialism, Weber acknowledged that capitalism involved the domination of value rationality by instrumental rationality. This, in turn, leads to tension between social groups with divergent interests and manifests itself in political “attempts to increase or decrease the substantive regulation of social and economic life” (Brubaker, 1984, p. 43).

This difference between instrumental and value rationality and the tension between them is crucial but is masked by the use of the “value sphere” to include the economic and political realms. Brubaker argued that the latter are so different from the intellectual, cultural, aesthetic, and erotic spheres that to group them together is “misleading” since it “obscures crucial differences between the two groups” (1984, p. 85). As a result, the tensions of the modern world are often perceived as a clash between ultimate values, whereas it is more accurately expressed as the tension between “ultimate value commitments on the one hand and the requirements of successful economic and political action, requirements that are alien to *all* questions of ultimate value, on the other” (Brubaker, 1984, p. 85, emphasis in the original). The consequence is that “there is hardly any room for the cultivation of acosmic brotherliness, unless it is among strata who are economically carefree” (Weber, 1946, p. 357) so that “brotherly conduct, as a result, can flourish only in the *interstices* of the modern social order” (Brubaker, 1984, p. 86, emphasis in the original). However, the tensions between instrumental rationality and value rationality appear to the individual to be the same as those between the conflicting standards of value rationality and the choice between these “cannot itself be a rational one, for it is precisely criteria of rationality that must be chosen” (p. 87)^4^.

Brubaker argued that Weber's insistence on the distinction between facts and values obscured his moral ideals. These are based on the concept of meaning linked with rationality, applied not just to individual actions but to human life as a whole. A meaningful life has dignity and moral worth if individual actions are integrated according to certain fundamental values, constant and integral over time, which Weber summarized as “personality”^5^. However, the main threat to the development of such a personality is the insidious spread of instrumental rationality, a view not explicitly stated by Weber but which Brubaker claims is implicit in the structure of his moral thought. In this sense, instrumental rationality results in the individual pursuing their “*given* subjective wants” (Weber, 1922/1978, p. 26 emphasis added), rather than those derived from their values and meanings of life “forged into purposes and thereby translated into rational-teleological action” (Weber, 1975, p. 192). In response, Weber proposed an ethic of responsibility which involves an integration of value and instrumental rationality, so that the ultimate values from the former are pursued using the dispassionate analysis from the latter.

The implications of such changes in rationality have also been considered by other scholars. Fevre (2003, 2005) described how the spread of economic rationality (which can be understood as a type of instrumental rationality) has led to the “demoralization” of Western culture. Although earlier scholars such as Smith (1776) saw morality as something immutable, later scholars, for example Marx (1867), recognized how morality was changing as money “became value itself rather than an expression of value” (Fevre, 2003, p. 11). However, Fevre argued that classical theory took a wrong turning in accepting the dominance of instrumental rationality and ignoring the implications this had for morality. Wilson (2004) also analyzed the relationship between Weber's substantive and formal rationality and describes how the latter came to be prioritized, and Kalberg (1980) summarized Weber's argument from *The Protestant Ethic and the Spirit of Capitalism* (Weber, 1905) in the form of a transition from the value-rational action of devout Calvinists to means-end rational action in secular industrial society^6^.

Inevitably, such views are not undisputed. Suddaby et al. (2017), for example, challenged “the prevailing view of progressive rationality and disenchantment as set out in Max Weber's social theory and reproduced in organizational neo-institutionalism” (p. 1) and instead claimed that this is accompanied by the persistence of myth, magic and enchantment. Clegg (2005) applied Weberian concepts and methods to contemporary society and, noting that “rationalities are historically structured differently in varying periods, as different kinds of knowledge dominate” (p. 533), suggested that transformations are still most effective when based on value rationality. However, modern value rationality is based primarily on presentation of the self, exemplified by chief executives as charismatic visionaries, a world of consumption of “positional goods” (Hirsch, 1976), such as mobile phones, and McDonaldisation (Ritzer, 2004). In reaction, many people decide not to “play the game” and embrace new non-materialist values such as sustainability. In this regard, Clegg drew attention to the instrumental rationality behind the consumption of resources until Weber's last ton of fossilized fuel is used up, whilst at the same time noting that “in substantive or real terms this kind of rationality was idiocy” (Clegg, 2005, p. 541). Similarly, the literature on Corporate Social Responsibility (CSR) can be considered to be dominated by economic or instrumental rationality with scholars attempting to justify sustainable organizational behavior by means of financial arguments, chiefly in response to comments made by Friedman in 1970 (Brooks, 2010). However, doing so undermines the relevance of moral arguments and it can be argued that it is only by means of arguments based on value rationality that the critical potential of CSR will be realized.

### Work to date

These connections between neo-institutional theory and MacIntyre can be seen by the work already published in this area. For example, Moore and Grandy (2017) stated that “new institutional theory has adopted an assumption of organizations as amoral entities” (p. 2) and proposed that morality can be reintroduced by incorporating the work of MacIntyre. Whilst institutional theory acknowledges the central role played by practices, the definition commonly used lacks MacIntyre's richness and attention to moral considerations. Furthermore, although institutional logic's concept of legitimacy does provide a consideration of ethics, this is only from a ‘satisficing' perspective (Deephouse and Suchman, 2008). Moore and Grandy (2017) contended that institutional logics are intertwined with individual and collective *telos* and that legitimacy is associated with organizational purpose. In this respect, they differentiated between internal legitimacy, closely tied to practices, internal goods and identity, external legitimacy related more to external goods, and also moral legitimacy, which is dependent upon practices and the way in which they contribute to practitioners' and the community's *telos*. By such a consideration of MacIntyrean notions, they argued that institutional logics' lack of moral considerations can be addressed. However, it is clear from what has been said that these parallels can be explored further which is what I shall now endeavor to undertake.

### Bringing it together

In order to bring together MacIntyrean virtue ethics and the institutional logics perspective, differences in their definitions of institution need first to be addressed. Most obvious here is that institutions are at the societal level for institutional theory whilst at the organizational level for MacIntyre. For MacIntyre, an institution is the social bearer of practices (MacIntyre, 2007, p. 194) and so, in this sense, if the activities being carried out by an organization cannot be considered to be practices, then the organization cannot be considered a practice–institution combination. The institutional logics perspective also differentiates between practices and activities as described above, so bringing these concepts together, it can then be argued that practices are activities motivated by “the value for its own sake of some ethical, aesthetic, religious or other form of behavior” (Weber, 1922/1978).

One obvious area where MacIntyre's concepts can be used to augment the institutional logics perspective is the former's contention that there must be some *telos* to human life, whereas this aspect is almost completely lacking in the latter (Friedland excepted as noted above, for example Friedland, 2013a; Friedland and Arjaliès, 2019). Although the individual has goals within each institutional logic, this is a relatively minor factor within the framework. However, if these concepts are then combined, it can be argued that, since each of the individual institutional logics has its own driving rationality, a “logical or teleological consistency” that exercises a “power over man (Weber, 1920/1958, p. 324)” (Friedland, 2013b, p. 16), it is plausible then to suggest that there is a link to MacIntyre's *telos*. Everyday life is lived under the continual influence of multiple institutional logics, with the influence of each in any particular situation being influenced by its salience and availability, as described by the cross-functional model. The rationalities at the level of each of the institutional logics are internalized into the goals and schema of the individual via the process of institutionalization and so can be said to shape the goals of the individual within that situation. Putting this into the context of MacIntyre's child learning to play chess, for example, the child's choice between cheating to get the external goods of sweets or valuing playing for its own internal goods can be reformulated as the child being either swayed by instrumental rationality in the former case or by the value rationality of the aesthetic or intellectual value-sphere in the latter. Similarly, the two fishing crews often referred to by MacIntyre (1994, 1999a) can also be described in these terms so that the first is following the instrumental rationality of the market whereas the second is more influenced by the value rationality of the community. This is similar to Kay's consideration of professional and instrumental behavior when he considered the former to be associated with MacIntyre's practices more to do with the pursuit of internal goods whereas the latter was directed at the external good of making a profit in the market (Kay, 1997). Likewise (Knight, 2007, p. 164) stated that MacIntyre “had identified instrumental rationality predominantly with the bureaucratic state and managerial institutions of corporate capitalism” and similar linkages have also been made by other scholars (Dobson, 1999; Warneryd et al., 2005; Fisher and Byrne, 2012). Internal goods are those gained from engaging in practices, such as internal satisfaction, engagement with the community of practitioners, advancement of the standards of excellence and so on, whereas external goods are typically means to a further end. As such, it can be argued that internal goods can function as the reward of the individual as a result of their value rationality driven action, the fulfillment of the goals of the related institutional logic, its *telos*, together with an increase in its salience and the satisfaction of the associated values. In contrast, external goods are more to do with the achievement of goals driven by instrumental rationality. Consequently, the concept of *telos* and that of each institutional logic having its own value rationality can be argued to be linked.

It is clear, however, that this comparison should not be taken too far, since *telos* is concerned with man-as-he-could-be (what he “ought” to be) whereas rationality is concerned with man-as-is, which is one of the key arguments in *After Virtue*, since there can be no “ought” from “is” without a valid conception of *telos*. Indeed, MacIntyre criticized Weber as being an emotivist since “while he holds that an agent may be more or less rational in acting consistently with his values, the choice of any one particular evaluative stance or commitment can be no more rational than that of any other” (MacIntyre, 2007, p. 26). However, this view has been refuted by Tester (1999), who considered that MacIntyre misreads Weber's sociological project since Weber not only analyzed the condition of emotivism but also rejected it. In this regard, it may be that if the goals of the various institutional logics could be brought into alignment with each other and so form more of a common goal, then this can be argued to be more similar to MacIntyre's notion of *telos* guiding the individual's life. Indeed, this is what Weber summarized as “personality,” the ability to coordinate individual actions according to certain fundamental values, constant and integral over time, contributing to a meaningful life with dignity and moral worth. Nevertheless, for the current purposes of considering how MacIntyre's concepts and those of the institutional logics perspective can complement each other, it is sufficient to be aware of both the similarities and the differences.

It is assumed that current readers are familiar with the three-level framework of the virtues proposed by MacIntyre in *After Virtue* – those associated with the internal goods produced by practices housed within institutions, the narrative unity of a human life and moral tradition. Each of these can be reconsidered in the light of the concepts presented above as follows.

MacIntyre's first level of virtue was defined as:

“A virtue is an acquired human quality the possession and exercise of which tends to enable us to achieve those goods which are internal to practices and the lack of which effectively prevents us from achieving any such goods.” (MacIntyre, 2007, p. 191)

Thus, virtues can be considered to be aspects of the individual which enable and reinforce value rationality, including those which allow the individual to recognize and value the difference between internal and external goods. As well as being personal characteristics, such as *phronesis* or practical reasoning, honesty and courage considered by MacIntyre, these can also be associated with parts of the identity, goals and schema of the individual as depicted in the cross-level model. These are intimately linked to the institutional logics influential within any particular situation, which ties into the second of MacIntyre's levels, that of the narrative quest or unity of a human life:

“The virtues therefore are to be understood as those dispositions which…sustain us in the relevant kind of quest for the good, by enabling us to overcome the harms, dangers, temptations, and distractions which we encounter, and which will furnish us with increasing self-knowledge and increasing knowledge of the good.” (MacIntyre, 2007, p. 219)

Lutz (2012) considered this second level to be raising the focus from that of the individual in specific situations to that of their whole lives, where they move from one situation to another, acting out different social roles and so experiencing different influences. MacIntyre illustrated this negatively in terms of the compartmentalisation of the lives of power company executives (MacIntyre, 1999b) and their consequent lack of integrity across different aspects of their lives. He proposed that the whole person needs to be considered using “a concept of a self whose unity resides in the unity of a narrative which links birth to life to death as narrative beginning to middle to end” (MacIntyre, 2007, p. 205). This narrative is related to values and beliefs, with human actions being enacted narratives (MacIntyre, 2007, p. 211) and so there is a clear link here to the schema of the institutional logics perspective cross-level model. As the individual moves from one situation to another, they are influenced by different institutional logics with consequent changes in their activated identities, schema and goals. The dispositions sustaining them are then those enabling a degree of constancy between these differing scenarios with integrity being reflected in value rationality. The degree of commonality of the individual's identity, schema and goals throughout the differing institutional logics they function within will act against compartmentalisation and promote consistency, integrity and a unity of narrative supporting the achievement of their value-rational goals.

However, it is clear that this constancy also needs to be evident throughout the individual's life over time (Robson, 2015) and this can be provided by the concept of identity from institutional theory. Albert and Whetten (1985) proposed that one of the three aspects of identity was the provision of temporal continuity and, although they were discussing organizational identity, this aspect can also be argued to be applicable to the individual. In a related fashion, Weaver (2006) considered moral agents to have a moral identity which is strongly influenced by the prevailing institutional logics and he described how it is the stability, i.e., the enduring aspect, of non-market entities, such as the family and the community, which is important in providing this moral identity. MacIntyre (2007, p. 216–218) also considered identity to be an important concept in the narrative unity of a human life, dependent on social and historical factors—“the self has to find its moral identity in and through its membership in communities such as those of the family, the neighborhood, the city and the tribe” (MacIntyre, 2007, p. 221). Consequently, it is this moral identity, formed partly from interaction with the individual's institutional environment, which can provide the constancy and integrity of the individual over time, and so their narrative unity throughout their lifetime.

This then leads on to MacIntyre's third level of virtue, that of moral tradition:

“The virtues find their point and purpose… in sustaining those traditions which provide both practices and individual lives with their necessary historical context.” (MacIntyre, 2007, p. 223)

with tradition defined as “an historically extended, socially embodied argument, and an argument precisely in part about the goods which constitute that tradition” (MacIntyre, 2007, p. 222). This notion of tradition was further developed in *Whose Justice? Which Rationality?* (MacIntyre, 1988), where the relationship between virtue and practical rationality was refined, with both being intimately associated with tradition, negating the concept of an a-historic and abstract tradition-free rationality (p. 334).

It must be noted that MacIntyre's treatment of tradition is open to criticism. Horton and Mendus (Horton and Mendus, 1994, p. 13) stated that “MacIntyre's treatment of tradition... is also marked by a certain ambiguity or unclarity” whilst Porter (2003) points out that MacIntyre never defined the term throughout his works and that its focus changes over time. Thus, in *After Virtue* (2007), tradition played a role analogous to Aristotle's metaphysical biology, providing purpose and meaning to the narrative quest of individual lives. However, this emphasis changed in *Whose Justice, Which Rationality?* (1988) to one where tradition is considered to be guiding rationality until in the subsequent work *Three Rival Versions of Moral Enquiry* (1990), tradition was considered to be a form of moral inquiry. Consequently, Porter stated that “especially in his later works, MacIntyre moves between a wider concept of tradition as an overall social and moral orientation, and a more limited concept of a tradition as a focused scientific or moral inquiry” (Porter, 2003, p. 39). In contrast, it is interesting to note that tradition played almost no part in the later work *Dependent Rational Animals* (1999a). These changes in the treatment of tradition can be considered to be confusing, to say the least, with tradition changing from being a moral concept concerning virtue in *After Virtue* (2007) to an epistemic and linguistic concept concerning truth and rationality in *Whose Justice? Which Rationality?* (1988).

Nevertheless, it can be argued that the aspect of MacIntyre's concept of tradition where it is guiding rationality can be related to the way in which institutional theory presents tradition as the result of a process of “sedimentation,” whereby aspects of the prevailing culture over time become incorporated into tradition and so have an ongoing influence over future generations (Berger and Luckmann, 1966/1991, p. 85–89). In terms of the institutional logics perspective, culture can be seen as particular constellations of individual institutional logics, and so tradition can also be seen as sets of institutional logics which have arisen as a result of the sedimentation process. Consequently, MacIntyre's moral tradition can then be represented as those constellations of individual institutional logics which over time have promoted the particular identities, schema and goals which enable individuals to act in value-rational ways in order to achieve the internal goods of practices. This was exemplified by MacIntyre's critical argument in the first half of *After Virtue* where he described how concepts of the self and morality changed over time, with Aristotelian *telos* becoming replaced by divine will in the twelfth century, which then became displaced by the rationality of the Enlightenment (MacIntyre, 2007, p. 62). This can be related to the initial influence of the family and community institutional logics declining under the rise to dominance of a specific form of the religious institutional logic in Western Europe, which was then itself replaced by the emerging market institutional logic associated with the rise of capitalism.

### Taking it forwards

So far, I have emphasized the parallels between concepts from MacIntyre and neo-institutional theory, especially the institutional logics perspective. The major critique of the mainstream view of institutional logics concerning a lack of values can be addressed by bringing back notions of Weberian value and instrumental rationality which Friedland equated to his concept of the centrality of institutional substances. These can be compared to MacIntyre's view of *telos* and the differentiation of internal and external goods enabled by the virtues. However, this may still result in a resounding “So what?” from the reader and so it is important to take forward these ideas to demonstrate their application to current real-world examples.

Both MacIntyre and the institutional logics perspective agree that practices generate goods. However, the institutional logics perspective generally does not consider the nature of these goods, their different moral content or the role which they play within wider society. Friedland is a partial exception is this respect—he emphasized that each logic has its central institutional substances, “the god-terms of institutional life” (Friedland, 2013b, p. 19) akin to Aristotelian form, which provide the force for its value rationality. However, the MacIntyrean distinction between internal and external goods is the crucial addition, opening the way for the development of a typology of goods. This emphasizes that it is internal goods which contribute most to the *eudaimonia* of the practitioners and relegates external goods to the role of “merely” providing the supporting means by which this can be achieved. Of course, as MacIntyre insisted, both are important and so a balance in the pursuit of both is needed, but the emphasis on internal goods runs contrary to the often implicit assumptions of much of current theory, especially in the dominant economic and political arenas. To demonstrate the alternative nature of this position, a consequent depiction of the macro-level social environment will now be given. It is, of course, very generalized but is intended to serve as a high-level introduction to how such a view of the environment may appear.

I start with the generally accepted position, described above, that practices within meso-level organizations within the market logic generate goods. These can be both internal and external goods and it is the internal goods which contribute most to the *eudaimonia* of the practitioners at the micro-level. However, it is the external goods which are usually taken to be the most important and so form the primary focus of management, shareholders and the media. These external goods in the form of profits can not only be reinvested to enable the ongoing survival of the organization, but also provide the means to pay taxes to the state. This in turn enables the macro-level provision by the state of common goods such as education, transport infrastructure, justice and democracy, although this is not to say that common goods are not provided by other bodies. Similarly, salaries and wages paid by organizations to individuals enable the subsistence of the family and the consequent provision of internal goods to individuals within them. The salaries and wages also enable engagement in social and cultural activities within the community including the funding of professional and religious bodies, which may not only provide common goods but also internal goods via the practices housed within them.

This depiction is intended to show the importance of this typology of goods at the macro-level. Few would argue that the proper measure of the success of a family or a religious body is the amount of profit it makes, whilst this is taken for granted for commercial organizations. This demonstrates the acceptance of the purpose of an organization (in its wider sense) with respect to its ordering and provision of internal and external goods and is illuminated by the description of organizations offered by (Mutch, 2021, p. 14) as “bundles of practices given relatively enduring form...not just economic in nature [pursuing]…the substances that motivate them”. In this way, it can be argued that the primary purpose of commercial organizations within the market logic is the provision of external goods, that of the state is the provision of common goods and that of families, communities, religious bodies is the provision of internal goods via the practices which they house.

This is a simplified view but its aim is to show how a typology of goods combining concepts from MacIntyrean and the institutional logics perspective can throw aspects of society into sharp relief once the difference between internal and external goods is made the focus of debate. I am not denying that practices within commercial organizations also generate internal goods, indeed that is one of the arguments running throughout this work. Much of the political debate between left and right involves the purpose of commercial organizations and the role of the state and this can be recast in the form of not only the distribution of external goods but also the relative importance of internal and external goods. Whether commercial organizations should solely pursue profit (Friedman, 1962, 1970) or endeavor to meet the obligations of a wider array of stakeholders (Freeman et al., 2010) can be reinterpreted in part as whether their sole responsibility is the pursuit of external goods or whether a balance of internal and external goods is more appropriate. Additionally on a more practical level, the behavior of individual firms, organization types and across different sectors can be assessed in terms of the degree to which they encourage practices which enable their employees to achieve internal goods. Of course, this can be associated with the concept of the “McDonaldisation” of society (Ritzer, 2004), which is not surprising due to the common influence of Weber (again). However, an explicit acknowledgment of the differing roles of internal and external goods, the difference between them and the qualities required to distinguish between them can enhance multiple areas of academic study, such as business ethics, management education and the meaningfulness of work.

## Conclusion

The preceding arguments have hopefully demonstrated how MacIntyrean concepts can be fruitfully employed to contribute to neoinstitutional theory, a developing area of study criticized for lacking an adequate conception of values and morality. Drawing on the common roots of Weberian theory, it is clear that there are many parallels between them which enable concepts from one to be applied to the other. This was illustrated by a simple depiction of the market economy which, I contend, demonstrates how a combination of concepts from MacIntyre and the institutional logics perspective can be usefully brought together. It can be argued that MacIntyre's concepts are often at a level of abstraction which does not make it easy to see how they can be applied to real-world situations. My rudimentary depiction hopefully demonstrates how the typology of goods can be used to illuminate the market economy to shed light on how *eudaimonia* is fostered by the pursuit of internal goods supported by external goods, bringing a previously lacking moral perspective to the institutional logics perspective. In return, the different institutional logics and multi-level analysis of the institutional logics perspective provides a useful framework by means of which MacIntyre's concepts can be more easily demonstrated. As a result, I hope to have helped explore a small part of what more and what else MacIntyrean thought can contribute.

## Data availability statement

The original contributions presented in the study are included in the article/supplementary material, further inquiries can be directed to the corresponding authors.

## Author contributions

The author confirms being the sole contributor of this work and has approved it for publication.

## Conflict of interest

The author declares that the research was conducted in the absence of any commercial or financial relationships that could be construed as a potential conflict of interest.

## Publisher's note

All claims expressed in this article are solely those of the authors and do not necessarily represent those of their affiliated organizations, or those of the publisher, the editors and the reviewers. Any product that may be evaluated in this article, or claim that may be made by its manufacturer, is not guaranteed or endorsed by the publisher.

## Footnotes

^1^Morality is defined both in the broad sense of distinctions between right and wrong, good and bad, and in the narrower Aristotelian / MacIntyrean sense of that which enables human flourishing (MacIntyre, 2016).

^2^Similarly, Macintyre describes how “modernity partitions each human life into a variety of segments, each with its own norms and modes of behavior. So work is divided from leisure, private life from public, the corporate from the personal” (MacIntyre, 2007, p. 204).

^3^There is a direct comparison here with MacIntyre (2007, p. 187) stating that “bricklaying is not a practice; architecture is. Planting turnips is not a practice; farming is”.

^4^This is analogous to MacIntyre's view of the impossibility of an a-historic and abstract tradition-free rationality (MacIntyre, 1988, p. 334).

^5^There are obvious parallels here to MacIntyre's unity of human life, the second part of his framework of the virtues – that of the narrative quest (MacIntyre, 2007, pp. 208-219).

^6^Again, this is mirrored by MacIntyre's account in, where he describes how concepts of the self and morality changed over time, with Aristotelian *telos* becoming replaced by divine will in the twelfth century, which then became displaced by the rationality of the Enlightenment (MacIntyre, 2007, p. 62ff).
